# Prevalence of Urinary Incontinence and Its Impact on Quality of Life Among Women in Saudi Arabia: A Cross-Sectional Study

**DOI:** 10.7759/cureus.108964

**Published:** 2026-05-16

**Authors:** Manal A Alqahtani, Gofran M Albraheem, Hind M Faqeeh, Jawaher H Al-lugbi, Walaa K Hakami, Nuha S Alasmari

**Affiliations:** 1 Obstetrics and Gynecology, Khamis Mushayt Maternity and Children Hospital, Abha, SAU; 2 Obstetrics and Gynecology, Al-Ahsa Maternity and Children's Hospital, Al-Ahsa, SAU; 3 Obstetrics and Gynecology, Khamis Mushayt Maternity and Children Hospital, Khamis Mushayt, SAU; 4 Obstetrics and Gynecology, Armed Forces Hospital Southern Region, Khamis Mushayt, SAU; 5 Obstetrics and Gynecology, Najran Maternity and Children's Hospital, Najran, SAU

**Keywords:** cross-sectional study, health-seeking behavior, pelvic floor disorders, prevalence, public health, quality of life, saudi arabia, stress urinary incontinence, urinary incontinence, women’s health

## Abstract

Background: Urinary incontinence (UI) is a common yet underrecognized condition among women and is associated with substantial physical, psychological, and social consequences. Despite its significant impact on quality of life, many affected women do not seek medical care because of embarrassment, stigma, or misconceptions regarding the condition. Limited data are available regarding the burden of UI and its effect on the quality of life among women in Saudi Arabia.

Objective: To assess the prevalence of urinary incontinence and evaluate its impact on quality of life among women in Saudi Arabia.

Methods: A descriptive cross-sectional study was conducted among women residing in Saudi Arabia using a structured self-administered electronic questionnaire. Adult women aged 18 years and older were included, while pregnant women and those with neurological disorders affecting bladder function were excluded. Data collected included sociodemographic characteristics, obstetric and medical history, urinary incontinence characteristics, quality of life impact, and health-seeking behavior. Quality of life was assessed using Likert-scale responses across multiple domains. Data were analyzed using the Statistical Package for the Social Sciences (SPSS), version 26 (IBM Corp., Armonk, New York, USA), and associations between urinary incontinence and selected variables were evaluated using the chi-square test, with a p-value of less than 0.05 considered statistically significant.

Results: A total of 442 women participated in the study. Urinary incontinence was reported by 40.3% (n = 178) of participants, while 59.7% (n = 264) reported no symptoms. Stress urinary incontinence was the most common subtype, reported by 38.2% (n = 68) of affected women, followed by mixed urinary incontinence in 32.6% (n = 58) and urge urinary incontinence in 23.6% (n = 42). The prevalence of UI increased with age, reaching 61.1% (n = 44) among women aged 50 years or older. Moderate to severe impairment in overall quality of life was reported by 61.8% (n = 110) of affected participants. Emotional well-being was moderately to severely affected in 65.2% (n = 116), while daily activities were moderately to severely affected in 61.8% (n = 110). Despite the significant burden, only 36.0% (n = 64) of affected women sought medical advice, whereas 64.0% (n = 114) did not seek healthcare. The most common barriers to seeking care were embarrassment, reported by 36.8% (n = 42), and the belief that UI is a normal condition, reported by 29.8% (n = 34).

Conclusion: Urinary incontinence is highly prevalent among women in Saudi Arabia and is associated with considerable impairment in quality of life. The condition particularly affects emotional well-being, daily functioning, and social participation. Despite its impact, healthcare-seeking behavior remains low because of social and cultural barriers. Increased awareness, early screening, and targeted public health interventions are essential to improve recognition, management, and quality of life outcomes among affected women.

## Introduction

Urinary incontinence (UI) is defined as the involuntary leakage of urine and is recognized as a common yet often underreported condition affecting women worldwide [1,2]. It is not a life-threatening disorder; however, it has substantial physical, psychological, social, and economic consequences that significantly impair quality of life. The condition can be classified into several types, including stress urinary incontinence, urge urinary incontinence, and mixed urinary incontinence, each with different underlying mechanisms and clinical presentations [3,4].

UI is particularly prevalent among women due to anatomical, hormonal, and reproductive factors [1-3]. Pregnancy, childbirth, especially vaginal delivery, aging, obesity, and chronic medical conditions such as diabetes mellitus are well-established risk factors. These factors contribute to weakening of the pelvic floor muscles and altered bladder function, increasing susceptibility to urinary leakage [3,4].

Globally, urinary incontinence affects a significant proportion of women, with prevalence estimates varying widely depending on population characteristics and study methodology [4-6]. Despite its high prevalence, many affected women do not seek medical care due to embarrassment, lack of awareness, or the belief that UI is a normal consequence of aging or childbirth. This underreporting contributes to delayed diagnosis and inadequate management [2,5]. In Saudi Arabia, urinary incontinence remains a largely unexplored topic. Cultural and social factors may further influence reporting patterns and healthcare-seeking behavior, potentially increasing the hidden burden of the disease.

The impact of urinary incontinence extends beyond physical symptoms. It has been associated with reduced quality of life, including limitations in daily activities, social withdrawal, emotional distress, anxiety, and depression. In some cases, it may also affect occupational performance and sexual health, further contributing to reduced well-being [5-10].

Given the high prevalence and multifaceted impact of urinary incontinence, particularly among women in reproductive and middle to older age groups, it is essential to assess its burden in specific populations. Understanding the extent of its impact on the quality of life among Saudi women can provide valuable insights for healthcare planning, early intervention strategies, and public health awareness programs. Therefore, this study aims to evaluate the prevalence of urinary incontinence and its effect on quality of life among women in Saudi Arabia using a cross-sectional design.

## Materials and methods

Study design and setting

This study was designed and reported in accordance with standard methodological approaches for cross-sectional research. A descriptive cross-sectional study was conducted among women residing in Saudi Arabia. Data were collected over a defined study period using a structured, self-administered questionnaire distributed electronically via online platforms. This design was chosen to estimate the prevalence of urinary incontinence and assess its impact on quality of life at a single point in time.

Study population and eligibility criteria

The study targeted adult women living in Saudi Arabia. Inclusion criteria were women aged 18 years and above who were able to read and understand the questionnaire and consented to participate. Exclusion criteria included women who were pregnant at the time of the study, those with known neurological disorders affecting bladder function, or those who provided incomplete responses.

Sample size and sampling technique

The sample size was calculated using a single proportion formula based on an expected prevalence of urinary incontinence of approximately 40%, a 95% confidence level, and a 5% margin of error. The minimum required sample size was estimated to be 369 participants; this was increased to account for potential non-response. A non-probability convenience sampling technique was used to recruit participants through social media and online distribution channels.

Data collection tool

Data were collected using a structured questionnaire developed based on previously validated instruments assessing urinary incontinence and quality of life. The questionnaire consisted of multiple-choice questions and was divided into five sections covering sociodemographic characteristics, including age, marital status, education, employment, and body mass index; obstetric and medical history, including gravidity, parity, mode of delivery, chronic diseases, and history of pelvic surgery; urinary incontinence characteristics, including presence, frequency, type, severity, and duration; the impact of urinary incontinence on quality of life across multiple domains, including daily activities, social life, emotional well-being, physical activity, sleep, and sexual life; and coping strategies and health-seeking behavior (see Appendix). The questionnaire was initially prepared in English and subsequently translated into Arabic. A pilot test was conducted on a small group of participants (n ≈ 15) to ensure clarity and comprehensibility, and minor modifications were made accordingly. Responses obtained from the pilot sample were excluded from the final analysis.

Outcome measures

The primary outcome was the presence of urinary incontinence, defined as self-reported involuntary leakage of urine. Secondary outcomes included the frequency, type, and severity of urinary incontinence, as well as its impact on quality of life. Quality of life was assessed using a Likert-scale response format (not at all, slightly, moderately, severely), with scores ranging from 0 to 3. An overall quality of life impact score was calculated and categorized into no impact, mild, moderate, and severe.

Data collection procedure

The questionnaire was distributed electronically using an online survey platform. Participation was voluntary, and informed consent was obtained at the beginning of the survey. The survey was anonymous, and no identifying information was collected. Data were collected over a predefined period until the target sample size was reached.

Statistical analysis

Data were entered and analyzed using the Statistical Package for the Social Sciences (SPSS), version 26 (IBM Corp., Armonk, New York, USA) or later. Descriptive statistics were used to summarize the data. Categorical variables were presented as frequencies and percentages. The prevalence of urinary incontinence was calculated as a proportion of the total sample.

## Results

Demographic and clinical characteristics

A total of 442 women participated in this study. The largest proportion of participants was aged 20-29 years (n = 132, 29.9%), followed by 30-39 years (n = 118, 26.7%). Married women constituted the majority of the sample (n = 268, 60.6%), while 124 (28.1%) were single. In terms of education, nearly half had a university degree (n = 214, 48.4%). Regarding body mass index, 152 participants (34.4%) were overweight, and 126 (28.5%) were obese (Table 1).

**Table 1:** Sociodemographic characteristics of the study participants. This table presents the sociodemographic characteristics of the study participants, including age distribution, marital status, educational level, employment status, and body mass index (BMI), with corresponding frequencies and percentages (n = 442).

Variable	Category	Frequency (n)	Percentage (%)
Age	<20	28	6.3
	20–29	132	29.9
	30–39	118	26.7
	40–49	92	20.8
	≥50	72	16.3
Marital status	Single	124	28.1
	Married	268	60.6
	Divorced	28	6.3
	Widowed	22	5.0
Education	Primary or less	46	10.4
	Secondary	128	29.0
	University	214	48.4
	Postgraduate	54	12.2
Employment	Student	102	23.1
	Employed	168	38.0
	Unemployed	54	12.2
	Housewife	118	26.7
BMI	Underweight	18	4.1
	Normal	146	33.0
	Overweight	152	34.4
	Obese	126	28.5

With respect to obstetric history, 130 women (29.4%) reported no previous pregnancies, while 92 (20.8%) had five or more. Vaginal delivery was reported by 178 participants (40.3%), whereas 102 (23.1%) had cesarean sections. The majority had no chronic diseases (n = 328, 74.2%), and 68 (15.4%) reported a history of pelvic surgery (Table 2).

**Table 2:** Obstetric and medical history of the study participants This table summarizes the obstetric and medical history of the study participants, including gravidity, number of deliveries, mode of delivery, presence of chronic diseases, and history of pelvic surgery, with corresponding frequencies and percentages (n = 442).

Variable	Category	Frequency (n)	Percentage (%)
Gravidity	0	130	29.4
	1–2	118	26.7
	3–4	102	23.1
	≥5	92	20.8
Deliveries	0	142	32.1
	1–2	124	28.1
	3–4	96	21.7
	≥5	80	18.1
Mode of delivery	Vaginal	178	40.3
	Cesarean	102	23.1
	Both	64	14.5
	N/A	98	22.2
Chronic disease	Diabetes	52	11.8
	Hypertension	38	8.6
	Both	24	5.4
	None	328	74.2
Pelvic surgery	Yes	68	15.4
	No	374	84.6

Prevalence and characteristics of urinary incontinence

Urinary incontinence was reported by 178 participants (40.3%), while 264 (59.7%) reported no symptoms. Among affected women, leakage occurred several times per week in 54 (30.3%) and daily in 44 (24.7%). Stress urinary incontinence was the most common type (n = 68, 38.2%), followed by mixed incontinence (n = 58, 32.6%) and urge incontinence (n = 42, 23.6%).

Regarding severity, most participants reported leaking small amounts (n = 64, 36.0%) or drops (n = 52, 29.2%). The duration of symptoms was most commonly one to three years (n = 58, 32.6%), followed by six to 12 months (n = 42, 23.6%) and more than three years (n = 42, 23.6%) (Table 3).

**Table 3:** Characteristics of urinary incontinence (UI) among affected participants. This table presents the characteristics of urinary incontinence among affected participants, including frequency of leakage, type of urinary incontinence, amount of leakage, and duration of symptoms, with corresponding frequencies and percentages (n = 178).

Variable	Category	Frequency (n)	Percentage (%)
Frequency of leakage	<1/week	38	21.3
	1/week	42	23.6
	Several/week	54	30.3
	Daily	44	24.7
Type of UI	Stress	68	38.2
	Urge	42	23.6
	Mixed	58	32.6
	Other	10	5.6
Amount of leakage	Drops	52	29.2
	Small	64	36.0
	Moderate	40	22.5
	Large	22	12.4
Duration	<6 months	36	20.2
	6–12 months	42	23.6
	1–3 years	58	32.6
	>3 years	42	23.6

Impact on quality of life

Urinary incontinence had a noticeable impact across multiple domains of quality of life. For daily activities, 62 participants (34.8%) reported moderate impact and 48 (27.0%) reported severe impact. Similarly, social life was moderately affected in 54 (30.3%) and severely affected in 44 (24.7%) (Table 4).

**Table 4:** Impact of urinary incontinence on quality of life domains among affected participants. This table illustrates the impact of urinary incontinence on different quality of life domains among affected participants, including daily activities, social life, work or study, emotional health, physical activity, sleep, and sexual life, categorized by level of impact (not at all, slightly, moderately, severely) with corresponding frequencies and percentages.

Domain	Not at all n (%)	Slightly n (%)	Moderately n (%)	Severely n (%)
Daily activities	22 (12.4)	46 (25.8)	62 (34.8)	48 (27.0)
Social life	28 (15.7)	52 (29.2)	54 (30.3)	44 (24.7)
Work/study	36 (20.2)	48 (27.0)	50 (28.1)	44 (24.7)
Emotional health	18 (10.1)	44 (24.7)	60 (33.7)	56 (31.5)
Physical activity	30 (16.9)	50 (28.1)	54 (30.3)	44 (24.7)
Sleep	40 (22.5)	52 (29.2)	46 (25.8)	40 (22.5)
Sexual life	52 (29.2)	36 (20.2)	42 (23.6)	48 (27.0)

Work or study performance was moderately affected in 50 participants (28.1%) and severely in 44 (24.7%). Emotional well-being showed one of the highest burdens, with 60 participants (33.7%) reporting moderate impact and 56 (31.5%) reporting severe impact. Physical activity was moderately affected in 54 (30.3%) and severely in 44 (24.7%), while sleep disturbance was reported as moderate in 46 (25.8%) and severe in 40 (22.5%). Sexual life was not affected in 52 participants (29.2%), whereas 48 (27.0%) reported a severe impact.

Overall quality of life and coping behavior

Overall, urinary incontinence had a moderate impact on quality of life in 62 participants (34.8%) and a severe impact in 48 (27.0%), while 48 (27.0%) reported mild impact and 20 (11.2%) reported no impact. Regarding coping strategies, 52 participants (29.2%) reported occasional use of protective pads, 48 (27.0%) reported frequent use, and 42 (23.6%) reported always using them. Despite this, only 64 women (36.0%) sought medical advice, whereas 114 (64.0%) did not seek care. Among those who did not seek medical help, the most common reason was embarrassment (n = 42, 36.8%), followed by the belief that the condition is normal (n = 34, 29.8%), lack of awareness (n = 24, 21.1%), and limited access to healthcare (n = 14, 12.3%) (Table 5).

**Table 5:** Coping strategies, health-seeking behavior, and barriers to seeking medical care among women with urinary incontinence. This table presents coping strategies and health-seeking behavior among women with urinary incontinence, including patterns of pad use and whether medical advice was sought. It also summarizes the reported barriers among participants who did not seek medical care, including embarrassment, perception that urinary incontinence is normal, lack of awareness, and access-related issues, with corresponding frequencies and percentages.

Variable	Category	Frequency (n)	Percentage (%)
Pad use	Never	36	20.2
	Occasionally	52	29.2
	Frequently	48	27.0
	Always	42	23.6
Sought medical help	Yes	64	36.0
	No	114	64.0
Reasons for not seeking medical help	Embarrassment	42	36.8
	Think it is normal	34	29.8
	Lack of awareness	24	21.1
	Access issues	14	12.3

Association between age and urinary incontinence

The prevalence of urinary incontinence increased with age. Among women younger than 30 years, 42 (26.0%) reported urinary incontinence, compared with 48 (40.7%) in the 30-39 age group, 44 (47.8%) in the 40-49 age group, and 44 (61.1%) among those aged 50 years or older. Conversely, the proportion of women without urinary incontinence decreased with increasing age, from 118 (74.0%) in those under 30 years to 28 (38.9%) in those aged 50 years or above.

## Discussion

This study assessed the prevalence of UI and its impact on quality of life among women in Saudi Arabia. The findings demonstrate that UI is a common condition with substantial physical, social, and psychological consequences, yet it remains underreported and undertreated.

The prevalence observed in this study is consistent with findings from previous studies conducted in Saudi Arabia and other regional settings, confirming that UI represents an important public health concern among women [2,6]. The relatively high prevalence may be related to the presence of recognized risk factors within the study population, particularly multiparity, obesity, and advancing age.

A clear association between increasing age and the occurrence of UI was identified. This finding is in agreement with the existing literature and may be explained by age-related weakening of pelvic floor muscles, hormonal changes, and the increasing prevalence of chronic medical conditions among older women [11-15]. Obstetric factors also appeared to contribute significantly to the development of UI. Multiple pregnancies and vaginal deliveries are well-established contributors to pelvic floor dysfunction and bladder control impairment. In addition, obesity may increase intra-abdominal pressure, thereby exacerbating urinary leakage symptoms [3,4].

Stress urinary incontinence emerged as the most frequently reported subtype, followed by mixed and urge urinary incontinence. This pattern is consistent with global epidemiological studies, where stress UI is generally the predominant type among women, especially those with a history of childbirth [4-7]. The findings further indicate that UI has a substantial negative impact on multiple domains of quality of life, including daily functioning, emotional well-being, social participation, physical activity, sleep quality, and occupational performance. Emotional health appeared to be particularly affected, emphasizing that the burden of UI extends beyond physical symptoms and may contribute to psychological distress, anxiety, and reduced self-esteem [4-7].

Social functioning was also negatively influenced, which may reflect the cultural and social sensitivities surrounding UI, particularly in conservative communities where embarrassment and fear of stigma may limit participation in social activities. Similarly, the effect on work and academic performance highlights the broader functional burden associated with the condition [4-10].

The impact on sleep and physical activity further suggests that UI can interfere with overall well-being and lifestyle. Sexual health was also affected among many participants, although underreporting in this domain is possible because of the sensitive nature of the topic and cultural reluctance to discuss intimate concerns openly [2,3,6].

Despite the significant burden associated with UI, healthcare-seeking behavior remained relatively low. Many women did not seek medical advice despite experiencing symptoms that affected their quality of life. Embarrassment, misconceptions that UI is a normal consequence of aging or childbirth, and limited awareness about available treatment options were identified as major barriers to seeking care. These findings emphasize the ongoing influence of stigma and cultural perceptions on healthcare utilization [2-7].

The findings of this study have important clinical and public health implications. Routine screening for UI in primary healthcare settings may facilitate earlier identification and management, particularly among high-risk groups such as older, multiparous, and obese women. In addition, educational initiatives are needed to improve awareness that UI is a treatable medical condition rather than an inevitable part of aging or childbirth. Healthcare professionals should also be encouraged to initiate sensitive discussions regarding UI to reduce stigma and improve patient disclosure.

Strengths and limitations

This study has several strengths, including a relatively large sample size (n = 442) and a comprehensive assessment of both clinical characteristics and quality of life domains. However, certain limitations should be acknowledged. The use of a convenience sampling method may limit the generalizability of the findings. Additionally, data were self-reported, which may introduce recall bias or underreporting due to the sensitive nature of the condition. Finally, the cross-sectional design precludes establishing causal relationships.

## Conclusions

This study demonstrates that urinary incontinence is a common and significant health problem among women in Saudi Arabia and is associated with considerable impairment in quality of life. The findings show that urinary incontinence negatively affects multiple aspects of daily living, particularly emotional well-being, social functioning, and physical activities. It is also associated with established risk factors such as advancing age, multiparity, vaginal delivery, and obesity. Despite this substantial burden, healthcare-seeking behavior remains low due to embarrassment, stigma, and misconceptions that urinary incontinence is a normal part of aging or childbirth.

These findings highlight the need for improved awareness, early identification, and appropriate management of urinary incontinence in clinical practice. Public health strategies should focus on reducing stigma, enhancing patient education, and encouraging timely medical consultation. Integrating routine screening for urinary incontinence into primary healthcare services may help promote earlier diagnosis and better outcomes. Overall, urinary incontinence remains an underrecognized condition with important physical, psychological, and social consequences, and addressing both clinical and sociocultural barriers is essential to improve quality of life among affected women.

## Appendices

Questionnaire

Sociodemographic and Clinical Data

1. Age (<20, 20-29, 30-39, 40-49, ≥50)

2. Marital status (Single, Married, Divorced, Widowed)

3. Education level (Primary or less, Secondary, University, Postgraduate)

4. Employment status (Student, Employed, Unemployed, Housewife)

5. BMI category (Underweight, Normal, Overweight, Obese)

6. Gravidity (0, 1-2, 3-4, ≥5)

7. Number of deliveries (0, 1-2, 3-4, ≥5)

8. Mode of delivery (Vaginal, Cesarean, Both, N/A)

9. Chronic disease history (Diabetes, Hypertension, Both, None)

10. History of pelvic surgery (Yes/No)

Urinary Incontinence Characteristics

11. Presence of urinary incontinence (Yes/No)

12. Frequency of leakage (<1/week, 1/week, Several/week, Daily)

13. Type of urinary incontinence (Stress, Urge, Mixed, Other)

14. Amount of leakage (Drops, Small, Moderate, Large)

15. Duration of symptoms (<6 months, 6-12 months, 1-3 years, >3 years)

Quality of Life Assessment

Participants rated the impact of urinary incontinence on the following domains using a 4-point Likert scale (0 = Not at all, 1 = Slightly, 2 = Moderately, 3 = Severely):

Daily activities

Social life

Work/study performance

Emotional well-being

Physical activity

Sleep quality

Sexual life

Coping and Healthcare-Seeking Behavior

16. Use of protective pads (Never, Occasionally, Frequently, Always)

17. Sought medical advice (Yes/No)

18. If no, reason for not seeking care (Embarrassment, Belief condition is normal, Lack of awareness, Access issues)

Quality of Life Scoring

Total QoL scores were categorized as:

No impact

Mild impact

Moderate impact

Severe impact 
